# μ-Decane­dioato-bis­[aqua­bis­(1,10-phenanthroline-κ^2^ *N*,*N*′)manganese(II)] dinitrate–sebacic acid–water (1/1/2)

**DOI:** 10.1107/S1600536810051615

**Published:** 2010-12-15

**Authors:** Ye-Qiang Feng, Jian Long, Juan Li

**Affiliations:** a Key Laboratory for Information Systems of Mountainous Areas and Protection of the Economical Environment of Guizhou Province, Guizhou Normal University, Guiyang 550001, People’s Republic of China; b College of Geographical and Environmental Sciences, Guizhou Normal University, Guiyang 550001, People’s Republic of China

## Abstract

In the title complex, [Mn_2_(C_10_H_16_O_4_)(C_12_H_8_N_2_)_4_(H_2_O)_2_](NO_3_)_2_·C_10_H_18_O_4_·2H_2_O, the asymmetric unit contains one-half of the centrosymmetric dinuclear complex cation, one uncoordinated water molecule, one-half of a free sebaic acid (decanedioic acid) molecule that is also completed by inversion symmetry, and one disordered nitrate anion [occupancy ratio 0.454 (4):0.544 (6)]. The Mn^II^ atoms are each octa­hedrally surrounded by four N atoms from two 1,10-phenanthroline (phen) ligands, one O atom from one carbonyl group of the bridging sebacate ligand and one O atom of a water mol­ecule. The crystal structure is stabilized by intermolecular O—H⋯O hydrogen bonds.

## Related literature

For applications of carb­oxy­lic metalorganic complexes, see: Lehn (2007 ▶); Wang *et al.* (2010 ▶); Fang & Zhang (2006 ▶). For related structures, see: Wei *et al.* (2002 ▶).
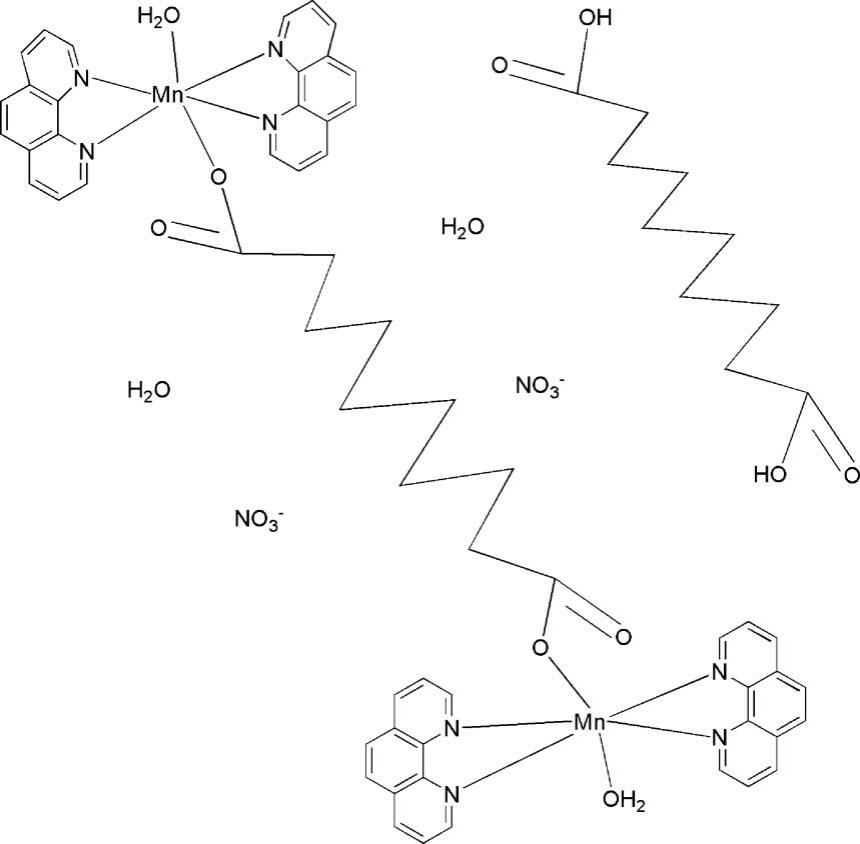


## Experimental

### 

#### Crystal data

[Mn_2_(C_10_H_16_O_4_)(C_12_H_8_N_2_)_4_(H_2_O)_2_](NO_3_)_2_·C_10_H_18_O_4_·2H_2_O
*M* *_r_* = 1429.00
Triclinic, 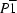

*a* = 11.6712 (3) Å
*b* = 12.5316 (3) Å
*c* = 12.8561 (3) Å
α = 76.678 (1)°
β = 66.845 (1)°
γ = 81.971 (1)°
*V* = 1679.79 (7) Å^3^
*Z* = 1
Mo *K*α radiation
μ = 0.46 mm^−1^
*T* = 296 K
0.58 × 0.33 × 0.13 mm

#### Data collection

Bruker APEXII diffractometer
Absorption correction: multi-scan (*SADABS*; Bruker, 2002 ▶) *T*_min_ = 0.837, *T*_max_ = 0.943
25302 measured reflections
7666 independent reflections
6144 reflections with *I* > 2σ(*I*)
*R*_int_ = 0.025

#### Refinement

*R*[*F* ^2^ > 2σ(*F* ^2^)] = 0.036
*wR*(*F* ^2^) = 0.085
*S* = 1.03
7666 reflections
449 parameters
H-atom parameters constrained
Δρ_max_ = 0.50 e Å^−3^
Δρ_min_ = −0.38 e Å^−3^

### 

Data collection: *APEX2* (Bruker, 2006 ▶); cell refinement: *SAINT* (Bruker, 2006 ▶); data reduction: *SAINT*; program(s) used to solve structure: *SHELXS97* (Sheldrick, 2008 ▶); program(s) used to refine structure: *SHELXL97* (Sheldrick, 2008 ▶); molecular graphics: *SHELXTL* (Sheldrick, 2008 ▶); software used to prepare material for publication: *SHELXTL*.

## Supplementary Material

Crystal structure: contains datablocks global, I. DOI: 10.1107/S1600536810051615/bx2333sup1.cif

Structure factors: contains datablocks I. DOI: 10.1107/S1600536810051615/bx2333Isup2.hkl

Additional supplementary materials:  crystallographic information; 3D view; checkCIF report

## Figures and Tables

**Table 1:** Hydrogen-bond geometry (Å, °)

*D*—H⋯*A*	*D*—H	H⋯*A*	*D*⋯*A*	*D*—H⋯*A*
O1*W*—H1*A*⋯O2^i^	0.85	1.83	2.6680 (16)	170
O2*W*—H2*D*⋯O6*A*^ii^	0.85	2.24	2.991 (6)	148
O2*W*—H2*D*⋯O7^ii^	0.85	2.22	3.020 (2)	157
O1*W*—H1*B*⋯O6*B*	0.89	1.87	2.710 (4)	156
O1*W*—H1*B*⋯O5*A*	0.89	1.99	2.830 (5)	156
O2*W*—H2*C*⋯O2	0.85	1.90	2.7396 (19)	170
O4—H4*C*⋯O2*W*	0.85	1.78	2.622 (2)	172

Symmetry codes: (i) 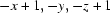; (ii) 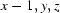.

**Fig. 1.**
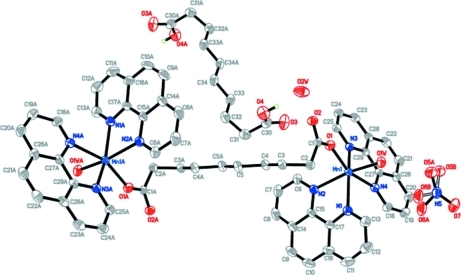
The molecular structure of the title complex, showing the atom-labelling scheme. Displacement ellipsoids are drawn at the 50% probability level, hydrogen atoms were omitted for clarity.

## supplementary crystallographic information

### Comment

The design and synthesis of carboxylic metal-organic complexes have been an increasing interest for many years owing to their potential practical applications, such as fluorescence, magnetism (Wang, *et al.*, 2010; Fang, *et al.*, 2006; Lehn, *et al.*, 2007). Interested in this field, herein, we report the crystal structure of a new manganese^II^ complex with the sebacic acid ligand. The asymmetric unit contains a centrosymmetric dinuclear complex cation, one water molecule, a free sebacic acid and one nitrato anion. The Mn atoms are each octahedrally surrounded by four N atoms from two phen ligands, one O atom from one carbonyl group of the bridging sebacato ligand and one O atom of water molecule. The basal Mn—O and Mn—N bond lengths fall in the range 2.0934 (11)–2.1466 (11); 2.2821 (13)–2.3480 (13)Å respectively. The axial Mn—N bond lengths fall in the range 2.2596 (13)–2.3027 (14) Å. The sebasic acid molecule is linear and all C—C and C—O bond lengths are of normal values. The sebacate anion acts as monodentate ligand . The crystal structures is stabilized by three and four intermolecular and intramolecular O—H···O hydrogen bonds respectively, Fig.1. The phen ligands make a dihedral angle of 77.4 (3)°.

### Experimental

All reagents were used as received without purified. The title compound was obtained by adding sebacic acid (1 mmol) and 1,10-phenanthroline(phen) (2 mmol) in fifty percent ethanol solution (20 ml), then Mn(NO_3_)_2 _(1 mmol) dissolved in distilled water (10 ml) is slowly dripped into above solution, mixed round for five hours, filtrated, and single crystals were obtained after one week.

### Refinement

All H atoms attached to C atoms and *O*(hydroxyl) atom were fixed geometrically and treated as riding with C—H = 0.97 Å (methylene) or 0.93 Å (aromatic) and O—H = 0.85 Å with *U*_iso_(H) = 1.2*U*_eq_(C) or *U*_iso_(H) = 1.5*U*_eq_(O). H atoms of water molecule were located in a difference Fourier map and included in the subsequent refinement using restraints (O—H= 0.82 (1) Å and H···H= 1.30 (2) Å) with *U*_iso_(H) = 1.5*U*_eq_(O). The atom O5A and O6A are disordered. They were modelled using a split model with refined population parameters [O5A/O5B=0.456 (4)/0.544 (4); O6A/O6B=0.456 (4)/0.544 (4)].

### Crystal data

**Table:**

[Mn_2_(C_10_H_16_O_4_)(C_12_H_8_N_2_)_4_(H_2_O)_2_](NO_3_)_2_·C_10_H_18_O_4_·2H_2_O	*Z* = 1
*M**_r_* = 1429.00	*F*(000) = 746
Triclinic, *P*1	*D*_x_ = 1.413 Mg m^−^^3^
Hall symbol: -P 1	Mo *K*α radiation, λ = 0.71073 Å
*a* = 11.6712 (3) Å	Cell parameters from 9906 reflections
*b* = 12.5316 (3) Å	θ = 1.7–27.6°
*c* = 12.8561 (3) Å	µ = 0.46 mm^−^^1^
α = 76.678 (1)°	*T* = 296 K
β = 66.845 (1)°	Block, colorless
γ = 81.971 (1)°	0.58 × 0.33 × 0.13 mm
*V* = 1679.79 (7) Å^3^	

### Data collection

**Table:**

Bruker APEXII diffractometer	7666 independent reflections
Radiation source: fine-focus sealed tube	6144 reflections with *I* > 2σ(*I*)
graphite	*R*_int_ = 0.025
ω scans	θ_max_ = 27.6°, θ_min_ = 1.7°
Absorption correction: multi-scan (*SADABS*; Bruker, 2002)	*h* = −15→14
*T*_min_ = 0.837, *T*_max_ = 0.943	*k* = −15→16
25302 measured reflections	*l* = −16→16

### Refinement

**Table:**

Refinement on *F*^2^	Primary atom site location: structure-invariant direct methods
Least-squares matrix: full	Secondary atom site location: difference Fourier map
*R*[*F*^2^ > 2σ(*F*^2^)] = 0.036	Hydrogen site location: inferred from neighbouring sites
*wR*(*F*^2^) = 0.085	H-atom parameters constrained
*S* = 1.03	*w* = 1/[σ^2^(*F*_o_^2^) + (0.030*P*)^2^ + 0.580*P*] where *P* = (*F*_o_^2^ + 2*F*_c_^2^)/3
7666 reflections	(Δ/σ)_max_ < 0.001
449 parameters	Δρ_max_ = 0.50 e Å^−^^3^
0 restraints	Δρ_min_ = −0.38 e Å^−^^3^

### Special details

**Table:**

Geometry. All e.s.d.'s (except the e.s.d. in the dihedral angle between two l.s. planes) are estimated using the full covariance matrix. The cell e.s.d.'s are taken into account individually in the estimation of e.s.d.'s in distances, angles and torsion angles; correlations between e.s.d.'s in cell parameters are only used when they are defined by crystal symmetry. An approximate (isotropic) treatment of cell e.s.d.'s is used for estimating e.s.d.'s involving l.s. planes.
Refinement. Refinement of *F*^2^ against ALL reflections. The weighted *R*-factor *wR* and goodness of fit *S* are based on *F*^2^, conventional *R*-factors *R* are based on *F*, with *F* set to zero for negative *F*^2^. The threshold expression of *F*^2^ > σ(*F*^2^) is used only for calculating *R*-factors(gt) *etc*. and is not relevant to the choice of reflections for refinement. *R*-factors based on *F*^2^ are statistically about twice as large as those based on *F*, and *R*- factors based on ALL data will be even larger.

### Fractional atomic coordinates and isotropic or equivalent isotropic displacement parameters (Å^2^)

**Table:**

	*x*	*y*	*z*	*U*_iso_*/*U*_eq_	Occ. (<1)
Mn1	0.70121 (2)	0.230330 (18)	0.31787 (2)	0.03465 (8)	
O1	0.53707 (11)	0.15166 (9)	0.36166 (10)	0.0456 (3)	
O2	0.38982 (10)	0.03550 (9)	0.41943 (10)	0.0441 (3)	
O1W	0.68822 (11)	0.16789 (10)	0.49202 (10)	0.0474 (3)	
H1A	0.6574	0.1060	0.5275	0.071*	
H1B	0.7441	0.1750	0.5212	0.071*	
N1	0.88153 (13)	0.12483 (11)	0.24456 (12)	0.0423 (3)	
N2	0.74524 (13)	0.24656 (11)	0.12571 (12)	0.0407 (3)	
N3	0.58719 (12)	0.39148 (10)	0.33027 (11)	0.0370 (3)	
N4	0.82861 (12)	0.36202 (10)	0.31342 (11)	0.0381 (3)	
C1	0.50113 (15)	0.05984 (12)	0.36745 (13)	0.0339 (3)	
C2	0.59493 (16)	−0.02534 (14)	0.31001 (15)	0.0413 (4)	
H2A	0.6080	−0.0840	0.3690	0.050*	
H2B	0.6740	0.0081	0.2641	0.050*	
C3	0.55376 (16)	−0.07434 (13)	0.23259 (14)	0.0409 (4)	
H3A	0.6146	−0.1319	0.2030	0.049*	
H3B	0.4746	−0.1076	0.2785	0.049*	
C4	0.53961 (18)	0.00960 (14)	0.13196 (14)	0.0453 (4)	
H4A	0.6167	0.0470	0.0894	0.054*	
H4B	0.4740	0.0640	0.1615	0.054*	
C5	0.50857 (18)	−0.04110 (14)	0.05008 (15)	0.0478 (4)	
H5A	0.5750	−0.0947	0.0199	0.057*	
H5B	0.4325	−0.0799	0.0933	0.057*	
C6	0.67555 (19)	0.30097 (15)	0.06869 (16)	0.0524 (4)	
H6A	0.5980	0.3311	0.1103	0.063*	
C7	0.7121 (2)	0.31556 (18)	−0.05024 (18)	0.0651 (6)	
H7A	0.6596	0.3537	−0.0867	0.078*	
C8	0.8255 (2)	0.27326 (18)	−0.11219 (17)	0.0654 (6)	
H8A	0.8516	0.2828	−0.1919	0.078*	
C9	1.0253 (2)	0.1706 (2)	−0.11490 (19)	0.0692 (6)	
H9A	1.0561	0.1789	−0.1947	0.083*	
C10	1.0960 (2)	0.1170 (2)	−0.0567 (2)	0.0711 (7)	
H10A	1.1759	0.0903	−0.0974	0.085*	
C11	1.1218 (2)	0.04253 (18)	0.1314 (2)	0.0700 (6)	
H11A	1.2028	0.0157	0.0943	0.084*	
C12	1.0713 (2)	0.02624 (17)	0.2480 (2)	0.0679 (6)	
H12A	1.1171	−0.0116	0.2914	0.081*	
C13	0.94950 (19)	0.06706 (14)	0.30215 (18)	0.0543 (5)	
H13A	0.9145	0.0531	0.3823	0.065*	
C14	0.90359 (19)	0.21517 (16)	−0.05620 (16)	0.0539 (5)	
C15	0.85812 (16)	0.20248 (13)	0.06474 (14)	0.0412 (4)	
C16	1.05239 (17)	0.09953 (16)	0.06647 (19)	0.0556 (5)	
C17	0.93227 (15)	0.14120 (13)	0.12730 (15)	0.0418 (4)	
C18	0.94555 (16)	0.34774 (14)	0.30703 (16)	0.0472 (4)	
H18A	0.9895	0.2823	0.2891	0.057*	
C19	1.00663 (18)	0.42543 (16)	0.32574 (18)	0.0558 (5)	
H19A	1.0888	0.4114	0.3208	0.067*	
C20	0.94419 (19)	0.52166 (16)	0.35118 (17)	0.0550 (5)	
H20A	0.9829	0.5738	0.3652	0.066*	
C21	0.7496 (2)	0.64238 (14)	0.38036 (16)	0.0539 (5)	
H21A	0.7849	0.6969	0.3945	0.065*	
C22	0.6327 (2)	0.65874 (14)	0.38290 (15)	0.0522 (5)	
H22A	0.5887	0.7249	0.3979	0.063*	
C23	0.45241 (18)	0.59084 (15)	0.36366 (15)	0.0508 (4)	
H23A	0.4065	0.6569	0.3751	0.061*	
C24	0.40184 (18)	0.50752 (16)	0.34740 (16)	0.0524 (5)	
H24A	0.3222	0.5167	0.3455	0.063*	
C25	0.47149 (16)	0.40809 (14)	0.33364 (15)	0.0444 (4)	
H25A	0.4345	0.3505	0.3265	0.053*	
C26	0.82112 (17)	0.54230 (13)	0.35622 (14)	0.0449 (4)	
C27	0.76600 (15)	0.45891 (12)	0.33745 (13)	0.0368 (3)	
C28	0.57400 (17)	0.57670 (13)	0.36306 (14)	0.0431 (4)	
C29	0.63978 (15)	0.47586 (12)	0.34317 (13)	0.0359 (3)	
O3	0.36732 (19)	0.40660 (15)	0.14822 (16)	0.0929 (6)	
O4	0.28810 (17)	0.24951 (13)	0.16774 (15)	0.0837 (5)	
H4C	0.2729	0.2445	0.2389	0.126*	
C30	0.3445 (2)	0.34024 (18)	0.1066 (2)	0.0613 (5)	
C31	0.3730 (2)	0.35127 (18)	−0.01941 (19)	0.0663 (6)	
H31A	0.4293	0.2905	−0.0474	0.080*	
H31B	0.4147	0.4188	−0.0610	0.080*	
C32	0.2553 (2)	0.35228 (17)	−0.04290 (18)	0.0630 (5)	
H32A	0.2779	0.3504	−0.1236	0.076*	
H32B	0.2111	0.2868	0.0026	0.076*	
C33	0.1694 (2)	0.45277 (16)	−0.01428 (18)	0.0585 (5)	
H33A	0.1603	0.4618	0.0616	0.070*	
H33B	0.2089	0.5168	−0.0692	0.070*	
C34	0.0418 (2)	0.44959 (16)	−0.01543 (17)	0.0586 (5)	
H34A	0.0022	0.3852	0.0387	0.070*	
H34B	0.0503	0.4422	−0.0917	0.070*	
N5	0.91636 (17)	0.22595 (15)	0.58128 (14)	0.0582 (4)	
O5A	0.8094 (5)	0.2322 (5)	0.6183 (5)	0.0960 (12)	0.456 (4)
O6A	0.9670 (4)	0.1569 (6)	0.5070 (6)	0.0749 (10)	0.456 (4)
O5B	0.8208 (4)	0.2931 (4)	0.6291 (4)	0.0960 (12)	0.544 (4)
O6B	0.9028 (4)	0.1683 (5)	0.5265 (5)	0.0749 (10)	0.544 (4)
O7	1.00394 (16)	0.24493 (15)	0.60047 (16)	0.0858 (5)	
O2W	0.23404 (16)	0.21497 (15)	0.39055 (14)	0.0915 (6)	
H2C	0.2807	0.1610	0.4072	0.137*	
H2D	0.1631	0.2073	0.4453	0.137*	

### Atomic displacement parameters (Å^2^)

**Table:**

	*U*^11^	*U*^22^	*U*^33^	*U*^12^	*U*^13^	*U*^23^
Mn1	0.03696 (14)	0.03139 (12)	0.03438 (13)	−0.00389 (9)	−0.01003 (10)	−0.00884 (10)
O1	0.0437 (7)	0.0372 (6)	0.0569 (7)	−0.0086 (5)	−0.0166 (6)	−0.0115 (5)
O2	0.0378 (6)	0.0412 (6)	0.0486 (7)	−0.0086 (5)	−0.0098 (5)	−0.0078 (5)
O1W	0.0556 (7)	0.0497 (7)	0.0394 (6)	−0.0212 (6)	−0.0201 (6)	0.0020 (5)
N1	0.0450 (8)	0.0367 (7)	0.0458 (8)	−0.0011 (6)	−0.0159 (7)	−0.0119 (6)
N2	0.0434 (8)	0.0408 (7)	0.0376 (7)	−0.0067 (6)	−0.0122 (6)	−0.0098 (6)
N3	0.0390 (7)	0.0347 (7)	0.0356 (7)	−0.0025 (6)	−0.0115 (6)	−0.0080 (6)
N4	0.0399 (7)	0.0329 (7)	0.0387 (7)	−0.0045 (6)	−0.0112 (6)	−0.0062 (6)
C1	0.0390 (9)	0.0367 (8)	0.0284 (7)	−0.0053 (7)	−0.0160 (7)	−0.0031 (6)
C2	0.0416 (9)	0.0411 (9)	0.0445 (9)	−0.0006 (7)	−0.0184 (8)	−0.0115 (7)
C3	0.0465 (9)	0.0394 (8)	0.0372 (9)	−0.0020 (7)	−0.0138 (7)	−0.0115 (7)
C4	0.0561 (11)	0.0425 (9)	0.0383 (9)	−0.0007 (8)	−0.0173 (8)	−0.0116 (7)
C5	0.0605 (11)	0.0458 (9)	0.0404 (9)	−0.0028 (8)	−0.0208 (8)	−0.0113 (8)
C6	0.0587 (12)	0.0532 (11)	0.0496 (11)	−0.0059 (9)	−0.0248 (9)	−0.0084 (9)
C7	0.0830 (16)	0.0672 (13)	0.0547 (12)	−0.0159 (12)	−0.0371 (12)	−0.0025 (11)
C8	0.0900 (17)	0.0706 (14)	0.0373 (10)	−0.0302 (13)	−0.0197 (11)	−0.0062 (10)
C9	0.0680 (14)	0.0784 (15)	0.0474 (12)	−0.0259 (12)	0.0078 (11)	−0.0262 (11)
C10	0.0484 (12)	0.0748 (15)	0.0726 (15)	−0.0157 (11)	0.0129 (11)	−0.0374 (13)
C11	0.0425 (11)	0.0557 (12)	0.105 (2)	0.0026 (9)	−0.0155 (12)	−0.0288 (13)
C12	0.0599 (13)	0.0488 (11)	0.1039 (19)	0.0065 (10)	−0.0423 (14)	−0.0164 (12)
C13	0.0625 (12)	0.0409 (9)	0.0649 (12)	0.0035 (9)	−0.0297 (10)	−0.0137 (9)
C14	0.0610 (12)	0.0556 (11)	0.0406 (10)	−0.0240 (9)	−0.0041 (9)	−0.0155 (9)
C15	0.0445 (9)	0.0391 (8)	0.0377 (9)	−0.0137 (7)	−0.0064 (7)	−0.0124 (7)
C16	0.0410 (10)	0.0479 (10)	0.0722 (13)	−0.0077 (8)	−0.0057 (9)	−0.0252 (10)
C17	0.0391 (9)	0.0365 (8)	0.0476 (10)	−0.0092 (7)	−0.0068 (7)	−0.0165 (7)
C18	0.0418 (10)	0.0426 (9)	0.0553 (11)	−0.0039 (8)	−0.0160 (8)	−0.0087 (8)
C19	0.0467 (11)	0.0579 (11)	0.0655 (13)	−0.0139 (9)	−0.0218 (9)	−0.0092 (10)
C20	0.0591 (12)	0.0529 (11)	0.0585 (12)	−0.0211 (9)	−0.0220 (10)	−0.0105 (9)
C21	0.0723 (14)	0.0375 (9)	0.0505 (11)	−0.0141 (9)	−0.0160 (10)	−0.0121 (8)
C22	0.0748 (14)	0.0297 (8)	0.0444 (10)	−0.0015 (8)	−0.0135 (9)	−0.0095 (7)
C23	0.0590 (12)	0.0407 (9)	0.0433 (10)	0.0123 (8)	−0.0144 (9)	−0.0079 (8)
C24	0.0473 (10)	0.0580 (11)	0.0489 (11)	0.0091 (9)	−0.0194 (9)	−0.0092 (9)
C25	0.0437 (10)	0.0474 (9)	0.0423 (9)	−0.0001 (8)	−0.0158 (8)	−0.0109 (8)
C26	0.0565 (11)	0.0373 (8)	0.0386 (9)	−0.0133 (8)	−0.0127 (8)	−0.0057 (7)
C27	0.0447 (9)	0.0321 (8)	0.0295 (8)	−0.0070 (7)	−0.0089 (7)	−0.0042 (6)
C28	0.0558 (11)	0.0340 (8)	0.0320 (8)	0.0018 (7)	−0.0105 (7)	−0.0052 (7)
C29	0.0455 (9)	0.0295 (7)	0.0277 (7)	−0.0032 (6)	−0.0093 (7)	−0.0037 (6)
O3	0.1232 (16)	0.0853 (12)	0.0995 (13)	−0.0169 (11)	−0.0701 (12)	−0.0161 (10)
O4	0.1072 (14)	0.0677 (10)	0.0714 (11)	−0.0128 (10)	−0.0318 (10)	−0.0026 (9)
C30	0.0545 (12)	0.0572 (12)	0.0760 (15)	0.0069 (10)	−0.0327 (11)	−0.0111 (11)
C31	0.0625 (13)	0.0583 (12)	0.0690 (14)	−0.0039 (10)	−0.0136 (11)	−0.0145 (11)
C32	0.0810 (15)	0.0592 (12)	0.0493 (11)	−0.0185 (11)	−0.0212 (11)	−0.0086 (10)
C33	0.0711 (13)	0.0553 (11)	0.0552 (12)	−0.0178 (10)	−0.0295 (10)	−0.0034 (9)
C34	0.0749 (14)	0.0588 (11)	0.0511 (11)	−0.0239 (10)	−0.0311 (11)	−0.0017 (9)
N5	0.0613 (11)	0.0704 (11)	0.0484 (9)	−0.0119 (9)	−0.0267 (8)	−0.0056 (8)
O5A	0.0672 (16)	0.135 (4)	0.1006 (19)	0.035 (3)	−0.0375 (13)	−0.064 (3)
O6A	0.087 (3)	0.0818 (17)	0.084 (2)	−0.006 (3)	−0.053 (3)	−0.0313 (14)
O5B	0.0672 (16)	0.135 (4)	0.1006 (19)	0.035 (3)	−0.0375 (13)	−0.064 (3)
O6B	0.087 (3)	0.0818 (17)	0.084 (2)	−0.006 (3)	−0.053 (3)	−0.0313 (14)
O7	0.0767 (11)	0.1024 (13)	0.1040 (13)	−0.0243 (10)	−0.0488 (10)	−0.0304 (11)
O2W	0.0707 (11)	0.0983 (13)	0.0730 (11)	0.0261 (9)	−0.0143 (9)	0.0041 (9)

### Geometric parameters (Å, °)

**Table:**

Mn1—O1	2.0934 (11)	C13—H13A	0.9300
Mn1—O1W	2.1462 (11)	C14—C15	1.409 (2)
Mn1—N3	2.2596 (13)	C15—C17	1.436 (2)
Mn1—N2	2.2821 (13)	C16—C17	1.404 (2)
Mn1—N1	2.3027 (14)	C18—C19	1.397 (2)
Mn1—N4	2.3480 (13)	C18—H18A	0.9300
O1—C1	1.2556 (18)	C19—C20	1.357 (3)
O2—C1	1.2496 (18)	C19—H19A	0.9300
O1W—H1A	0.8516	C20—C26	1.402 (3)
O1W—H1B	0.8939	C20—H20A	0.9300
N1—C13	1.325 (2)	C21—C22	1.340 (3)
N1—C17	1.362 (2)	C21—C26	1.431 (3)
N2—C6	1.322 (2)	C21—H21A	0.9300
N2—C15	1.356 (2)	C22—C28	1.430 (2)
N3—C25	1.322 (2)	C22—H22A	0.9300
N3—C29	1.3654 (19)	C23—C24	1.361 (3)
N4—C18	1.324 (2)	C23—C28	1.403 (3)
N4—C27	1.361 (2)	C23—H23A	0.9300
C1—C2	1.511 (2)	C24—C25	1.397 (2)
C2—C3	1.529 (2)	C24—H24A	0.9300
C2—H2A	0.9700	C25—H25A	0.9300
C2—H2B	0.9700	C26—C27	1.411 (2)
C3—C4	1.516 (2)	C27—C29	1.434 (2)
C3—H3A	0.9700	C28—C29	1.405 (2)
C3—H3B	0.9700	O3—C30	1.194 (3)
C4—C5	1.522 (2)	O4—C30	1.317 (3)
C4—H4A	0.9700	O4—H4C	0.8500
C4—H4B	0.9700	C30—C31	1.497 (3)
C5—C5^i^	1.514 (3)	C31—C32	1.517 (3)
C5—H5A	0.9700	C31—H31A	0.9700
C5—H5B	0.9700	C31—H31B	0.9700
C6—C7	1.390 (3)	C32—C33	1.515 (3)
C6—H6A	0.9300	C32—H32A	0.9700
C7—C8	1.355 (3)	C32—H32B	0.9700
C7—H7A	0.9300	C33—C34	1.502 (3)
C8—C14	1.403 (3)	C33—H33A	0.9700
C8—H8A	0.9300	C33—H33B	0.9700
C9—C10	1.339 (3)	C34—C34^ii^	1.512 (4)
C9—C14	1.424 (3)	C34—H34A	0.9700
C9—H9A	0.9300	C34—H34B	0.9700
C10—C16	1.432 (3)	N5—O5A	1.147 (6)
C10—H10A	0.9300	N5—O6B	1.182 (5)
C11—C12	1.354 (3)	N5—O7	1.206 (2)
C11—C16	1.399 (3)	N5—O5B	1.329 (4)
C11—H11A	0.9300	N5—O6A	1.344 (7)
C12—C13	1.396 (3)	O2W—H2C	0.8500
C12—H12A	0.9300	O2W—H2D	0.8500
			
O1—Mn1—O1W	87.16 (5)	N2—C15—C14	122.20 (17)
O1—Mn1—N3	88.76 (5)	N2—C15—C17	117.93 (14)
O1W—Mn1—N3	102.95 (5)	C14—C15—C17	119.87 (16)
O1—Mn1—N2	91.66 (5)	C11—C16—C17	117.1 (2)
O1W—Mn1—N2	162.78 (5)	C11—C16—C10	124.2 (2)
N3—Mn1—N2	94.19 (5)	C17—C16—C10	118.7 (2)
O1—Mn1—N1	114.20 (5)	N1—C17—C16	122.60 (17)
O1W—Mn1—N1	92.32 (5)	N1—C17—C15	117.97 (15)
N3—Mn1—N1	153.19 (5)	C16—C17—C15	119.43 (17)
N2—Mn1—N1	72.51 (5)	N4—C18—C19	123.66 (17)
O1—Mn1—N4	157.92 (5)	N4—C18—H18A	118.2
O1W—Mn1—N4	86.23 (4)	C19—C18—H18A	118.2
N3—Mn1—N4	72.28 (5)	C20—C19—C18	118.94 (18)
N2—Mn1—N4	100.72 (5)	C20—C19—H19A	120.5
N1—Mn1—N4	87.11 (5)	C18—C19—H19A	120.5
C1—O1—Mn1	140.58 (11)	C19—C20—C26	119.83 (17)
Mn1—O1W—H1A	120.0	C19—C20—H20A	120.1
Mn1—O1W—H1B	126.1	C26—C20—H20A	120.1
H1A—O1W—H1B	105.3	C22—C21—C26	121.07 (17)
C13—N1—C17	117.86 (16)	C22—C21—H21A	119.5
C13—N1—Mn1	126.61 (13)	C26—C21—H21A	119.5
C17—N1—Mn1	114.38 (11)	C21—C22—C28	121.46 (16)
C6—N2—C15	118.10 (15)	C21—C22—H22A	119.3
C6—N2—Mn1	126.09 (12)	C28—C22—H22A	119.3
C15—N2—Mn1	115.62 (11)	C24—C23—C28	119.77 (16)
C25—N3—C29	117.81 (14)	C24—C23—H23A	120.1
C25—N3—Mn1	125.27 (11)	C28—C23—H23A	120.1
C29—N3—Mn1	116.76 (10)	C23—C24—C25	118.93 (17)
C18—N4—C27	117.69 (14)	C23—C24—H24A	120.5
C18—N4—Mn1	127.89 (11)	C25—C24—H24A	120.5
C27—N4—Mn1	113.65 (10)	N3—C25—C24	123.50 (17)
O2—C1—O1	122.44 (15)	N3—C25—H25A	118.3
O2—C1—C2	118.14 (14)	C24—C25—H25A	118.3
O1—C1—C2	119.42 (14)	C20—C26—C27	117.54 (16)
C1—C2—C3	112.98 (13)	C20—C26—C21	123.32 (16)
C1—C2—H2A	109.0	C27—C26—C21	119.14 (17)
C3—C2—H2A	109.0	N4—C27—C26	122.33 (15)
C1—C2—H2B	109.0	N4—C27—C29	118.19 (14)
C3—C2—H2B	109.0	C26—C27—C29	119.49 (15)
H2A—C2—H2B	107.8	C23—C28—C29	117.61 (16)
C4—C3—C2	113.25 (14)	C23—C28—C22	123.34 (16)
C4—C3—H3A	108.9	C29—C28—C22	119.05 (17)
C2—C3—H3A	108.9	N3—C29—C28	122.27 (15)
C4—C3—H3B	108.9	N3—C29—C27	118.00 (13)
C2—C3—H3B	108.9	C28—C29—C27	119.72 (15)
H3A—C3—H3B	107.7	C30—O4—H4C	110.9
C3—C4—C5	113.00 (14)	O3—C30—O4	123.4 (2)
C3—C4—H4A	109.0	O3—C30—C31	124.5 (2)
C5—C4—H4A	109.0	O4—C30—C31	112.14 (19)
C3—C4—H4B	109.0	C30—C31—C32	111.44 (18)
C5—C4—H4B	109.0	C30—C31—H31A	109.3
H4A—C4—H4B	107.8	C32—C31—H31A	109.3
C5^i^—C5—C4	114.19 (18)	C30—C31—H31B	109.3
C5^i^—C5—H5A	108.7	C32—C31—H31B	109.3
C4—C5—H5A	108.7	H31A—C31—H31B	108.0
C5^i^—C5—H5B	108.7	C33—C32—C31	112.29 (17)
C4—C5—H5B	108.7	C33—C32—H32A	109.1
H5A—C5—H5B	107.6	C31—C32—H32A	109.1
N2—C6—C7	123.5 (2)	C33—C32—H32B	109.1
N2—C6—H6A	118.3	C31—C32—H32B	109.1
C7—C6—H6A	118.3	H32A—C32—H32B	107.9
C8—C7—C6	118.9 (2)	C34—C33—C32	115.04 (17)
C8—C7—H7A	120.5	C34—C33—H33A	108.5
C6—C7—H7A	120.5	C32—C33—H33A	108.5
C7—C8—C14	120.04 (18)	C34—C33—H33B	108.5
C7—C8—H8A	120.0	C32—C33—H33B	108.5
C14—C8—H8A	120.0	H33A—C33—H33B	107.5
C10—C9—C14	120.9 (2)	C33—C34—C34^ii^	113.5 (2)
C10—C9—H9A	119.6	C33—C34—H34A	108.9
C14—C9—H9A	119.6	C34^ii^—C34—H34A	108.9
C9—C10—C16	122.0 (2)	C33—C34—H34B	108.9
C9—C10—H10A	119.0	C34^ii^—C34—H34B	108.9
C16—C10—H10A	119.0	H34A—C34—H34B	107.7
C12—C11—C16	120.2 (2)	O5A—N5—O6B	83.5 (3)
C12—C11—H11A	119.9	O5A—N5—O7	140.7 (3)
C16—C11—H11A	119.9	O6B—N5—O7	134.2 (3)
C11—C12—C13	119.1 (2)	O5A—N5—O5B	39.0 (3)
C11—C12—H12A	120.4	O6B—N5—O5B	118.1 (3)
C13—C12—H12A	120.4	O7—N5—O5B	107.6 (2)
N1—C13—C12	123.0 (2)	O5A—N5—O6A	114.4 (3)
N1—C13—H13A	118.5	O6B—N5—O6A	31.1 (2)
C12—C13—H13A	118.5	O7—N5—O6A	103.2 (3)
C8—C14—C15	117.25 (19)	O5B—N5—O6A	148.1 (3)
C8—C14—C9	123.68 (19)	H2C—O2W—H2D	105.9
C15—C14—C9	119.1 (2)		
			
O1W—Mn1—O1—C1	−86.07 (17)	Mn1—N2—C15—C14	−173.76 (12)
N3—Mn1—O1—C1	170.90 (17)	C6—N2—C15—C17	−178.53 (14)
N2—Mn1—O1—C1	76.74 (17)	Mn1—N2—C15—C17	6.19 (18)
N1—Mn1—O1—C1	5.20 (18)	C8—C14—C15—N2	−1.8 (2)
N4—Mn1—O1—C1	−158.77 (15)	C9—C14—C15—N2	177.29 (16)
O1—Mn1—N1—C13	−97.67 (15)	C8—C14—C15—C17	178.28 (16)
O1W—Mn1—N1—C13	−9.73 (15)	C9—C14—C15—C17	−2.7 (2)
N3—Mn1—N1—C13	115.53 (16)	C12—C11—C16—C17	2.2 (3)
N2—Mn1—N1—C13	178.56 (15)	C12—C11—C16—C10	−177.49 (19)
N4—Mn1—N1—C13	76.37 (15)	C9—C10—C16—C11	178.9 (2)
O1—Mn1—N1—C17	94.93 (11)	C9—C10—C16—C17	−0.8 (3)
O1W—Mn1—N1—C17	−177.14 (11)	C13—N1—C17—C16	0.0 (2)
N3—Mn1—N1—C17	−51.88 (17)	Mn1—N1—C17—C16	168.56 (13)
N2—Mn1—N1—C17	11.15 (10)	C13—N1—C17—C15	179.16 (15)
N4—Mn1—N1—C17	−91.04 (11)	Mn1—N1—C17—C15	−12.26 (17)
O1—Mn1—N2—C6	61.19 (14)	C11—C16—C17—N1	−2.2 (3)
O1W—Mn1—N2—C6	146.96 (16)	C10—C16—C17—N1	177.46 (16)
N3—Mn1—N2—C6	−27.69 (14)	C11—C16—C17—C15	178.62 (16)
N1—Mn1—N2—C6	176.08 (15)	C10—C16—C17—C15	−1.7 (2)
N4—Mn1—N2—C6	−100.43 (14)	N2—C15—C17—N1	4.2 (2)
O1—Mn1—N2—C15	−123.98 (11)	C14—C15—C17—N1	−175.80 (14)
O1W—Mn1—N2—C15	−38.2 (2)	N2—C15—C17—C16	−176.55 (14)
N3—Mn1—N2—C15	147.14 (11)	C14—C15—C17—C16	3.4 (2)
N1—Mn1—N2—C15	−9.09 (11)	C27—N4—C18—C19	1.3 (3)
N4—Mn1—N2—C15	74.40 (11)	Mn1—N4—C18—C19	−167.94 (14)
O1—Mn1—N3—C25	−14.49 (13)	N4—C18—C19—C20	−0.5 (3)
O1W—Mn1—N3—C25	−101.30 (13)	C18—C19—C20—C26	−1.1 (3)
N2—Mn1—N3—C25	77.08 (13)	C26—C21—C22—C28	0.8 (3)
N1—Mn1—N3—C25	135.54 (14)	C28—C23—C24—C25	1.8 (3)
N4—Mn1—N3—C25	177.00 (14)	C29—N3—C25—C24	1.3 (2)
O1—Mn1—N3—C29	160.80 (11)	Mn1—N3—C25—C24	176.52 (13)
O1W—Mn1—N3—C29	73.99 (11)	C23—C24—C25—N3	−3.2 (3)
N2—Mn1—N3—C29	−107.63 (11)	C19—C20—C26—C27	1.8 (3)
N1—Mn1—N3—C29	−49.17 (17)	C19—C20—C26—C21	−178.82 (18)
N4—Mn1—N3—C29	−7.71 (10)	C22—C21—C26—C20	179.20 (18)
O1—Mn1—N4—C18	146.84 (15)	C22—C21—C26—C27	−1.4 (3)
O1W—Mn1—N4—C18	73.97 (14)	C18—N4—C27—C26	−0.5 (2)
N3—Mn1—N4—C18	178.85 (15)	Mn1—N4—C27—C26	170.22 (12)
N2—Mn1—N4—C18	−90.14 (15)	C18—N4—C27—C29	179.24 (14)
N1—Mn1—N4—C18	−18.55 (14)	Mn1—N4—C27—C29	−10.02 (17)
O1—Mn1—N4—C27	−22.76 (19)	C20—C26—C27—N4	−1.0 (2)
O1W—Mn1—N4—C27	−95.63 (11)	C21—C26—C27—N4	179.60 (15)
N3—Mn1—N4—C27	9.26 (10)	C20—C26—C27—C29	179.24 (15)
N2—Mn1—N4—C27	100.26 (11)	C21—C26—C27—C29	−0.1 (2)
N1—Mn1—N4—C27	171.85 (11)	C24—C23—C28—C29	1.2 (3)
Mn1—O1—C1—O2	165.93 (12)	C24—C23—C28—C22	−178.06 (17)
Mn1—O1—C1—C2	−14.0 (2)	C21—C22—C28—C23	−179.27 (18)
O2—C1—C2—C3	50.5 (2)	C21—C22—C28—C29	1.5 (3)
O1—C1—C2—C3	−129.64 (15)	C25—N3—C29—C28	2.0 (2)
C1—C2—C3—C4	62.81 (19)	Mn1—N3—C29—C28	−173.70 (11)
C2—C3—C4—C5	175.50 (15)	C25—N3—C29—C27	−178.87 (14)
C3—C4—C5—C5^i^	178.9 (2)	Mn1—N3—C29—C27	5.47 (18)
C15—N2—C6—C7	−0.2 (3)	C23—C28—C29—N3	−3.2 (2)
Mn1—N2—C6—C7	174.50 (14)	C22—C28—C29—N3	176.11 (15)
N2—C6—C7—C8	−0.8 (3)	C23—C28—C29—C27	177.68 (14)
C6—C7—C8—C14	0.4 (3)	C22—C28—C29—C27	−3.1 (2)
C14—C9—C10—C16	1.5 (3)	N4—C27—C29—N3	3.4 (2)
C16—C11—C12—C13	0.0 (3)	C26—C27—C29—N3	−176.81 (14)
C17—N1—C13—C12	2.4 (3)	N4—C27—C29—C28	−177.37 (14)
Mn1—N1—C13—C12	−164.64 (14)	C26—C27—C29—C28	2.4 (2)
C11—C12—C13—N1	−2.4 (3)	O3—C30—C31—C32	−119.2 (2)
C7—C8—C14—C15	0.7 (3)	O4—C30—C31—C32	59.2 (2)
C7—C8—C14—C9	−178.27 (19)	C30—C31—C32—C33	65.7 (2)
C10—C9—C14—C8	179.2 (2)	C31—C32—C33—C34	−169.68 (17)
C10—C9—C14—C15	0.2 (3)	C32—C33—C34—C34^ii^	179.1 (2)
C6—N2—C15—C14	1.5 (2)		

Symmetry codes: (i) −*x*+1, −*y*, −*z*; (ii) −*x*, −*y*+1, −*z*.

### Hydrogen-bond geometry (Å, °)

**Table:**

*D*—H···*A*	*D*—H	H···*A*	*D*···*A*	*D*—H···*A*
O1W—H1A···O2^iii^	0.85	1.83	2.6680 (16)	170.
O2W—H2D···O6A^iv^	0.85	2.24	2.991 (6)	148.
O2W—H2D···O7^iv^	0.85	2.22	3.020 (2)	157.
O1W—H1B···O6B	0.89	1.87	2.710 (4)	156.
O1W—H1B···O5A	0.89	1.99	2.830 (5)	156.
O2W—H2C···O2	0.85	1.90	2.7396 (19)	170.
O4—H4C···O2W	0.85	1.78	2.622 (2)	172.

Symmetry codes: (iii) −*x*+1, −*y*, −*z*+1; (iv) *x*−1, *y*, *z*.
